# Isthmocele—a neglected cause of secondary infertility and implantation failure: A case report

**DOI:** 10.1002/ccr3.5853

**Published:** 2022-05-15

**Authors:** Elham Hosseini, Samaneh Aghajanpour, Nadia Zameni, Maryam Hafezi

**Affiliations:** ^1^ Department of Obstetrics and Gynecology Mousavi Hospital School of Medicine Zanjan University of Medical Sciences Zanjan Iran; ^2^ Department of Endocrinology and Female Infertility Reproductive Biomedicine Research Center Royan Institute for Reproductive Biomedicine ACECR Tehran Iran; ^3^ School of Medicine Shahid Beheshti University of Medical Sciences Tehran Iran

**Keywords:** implantation failure, isthmocele, secondary infertility

## Abstract

Isthmocele is myometrial scar tissue that develops after cesarean section delivery. In this case, other more prevalent pathologies delayed isthmocele diagnosis as the main cause of the patient's symptoms. Considering isthmocele is a fluid‐filled pouch‐like defect associated with infection caused by stagnant menstrual blood, its immunological aspects lead to implantation failure.

## Graphical abstract

Isthmocele's diagnosis had been neglected in the initial evaluation of the presented case. Physicians should pay more attention to isthmocele's impact mechanisms on secondary infertility, implantation failure, and associated attributes.

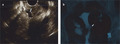

## 1 BACKGROUND

The long‐term effects of cesarean section (CS) delivery on the mother's health and future fertility are receiving more attention. One of the consequences of CS is the development of isthmocele. Isthmocele (its other names include niche or cesarean scar defect) is scar tissue that develops after CS delivery in the myometrium over the previous CS location. This defect has long‐term complications and consequences on the mothers' health, including menorrhagia, dysmenorrhea, and subsequent sub‐fertility/infertility.^1^, ^2^ Secondary infertility owing to isthmocele is thought to be caused by a decrease in follicular‐phase mucus quality, which hinders sperm storage and capacitation, and transfer, or even affects embryo implantation due to the stagnation of menstrual blood in the cervix.^3^ Isthmocele is graded into three categories based on ultrasound measurements and surface area (Base × Height)/2): grade 1 (≤15 mm^2^); grade 2 (16–25 mm^2^); and grade 3 (>25 mm^2^).^4^ There is another isthmocele classification by Ofili‐Yebovi D et al (2008) based on myometrial thickness at the defect site. It is measured by the ratio of the thickness of the defected myometrium to the thickness of the neighboring myometrium. A severe defect was defined as a ratio more than 50% and dehiscence as a ratio greater than 80%.^5^ The decision to treat isthmocele is based on different factors, including the extent of the defect, the severity of symptoms, future plans for childbearing, and the occurrence of secondary infertility.^2^ For instance, patients referring to the clinic with abnormal uterine bleeding, subsequent infertility, and pain in whom the residual myometrial thickness is >2–3 mm are preferably treated with hysteroscopic isthmoplasty, whereas patients presenting with the same symptoms but less residual myometrial thickness is commonly treated with the laparoscopic repair that is accompanied by hysteroscopic guidance.^6^

Most often, establishing isthmocele as the main cause of the patients' symptoms is delayed owing to other possible more common pathologies. However, since the incidence of cesarean delivery rate is increasing, it is essential to aware that isthmocele could be a sequela of CS and lead to secondary infertility.

## 2 CASE PRESENTATION

The couple with a history of two unsuccessful ICSI/ PGS/ET cycles was referred to infertility clinic. The 39‐year‐old woman has been suffering from secondary infertility for the past six years. The following is an overview of the couple's medical history and previous treatments: She had two spontaneous, full‐term pregnancies with no complications, for both of which cesarean sections were performed, due to cephalopelvic disproportion (CPD). The couple made the decision to use elective gender selection for female embryos in combination with ICSI for family balancing. The controlled ovarian stimulation and pickup yielded 15 oocytes. Based on the policy of the infertility clinic, all oocytes were frozen as to lower the risk of ovarian hyperstimulation syndrome (OHSS). Following endometrial preparation in the frozen‐thawed embryo transfer cycle, PGS was performed on embryos and then the desired embryos were transferred all of which failed to implant. The woman underwent another ovarian stimulation accompanied by ICSI the following year, in which once again all the embryos were frozen. The reason for embryo freezing was the thin and hyperechogenic pattern of the endometrium. Due to the couple's reluctance to undergo gender selection by PGS in this cycle, their frozen embryos were transferred in two separate embryo transfer cycles. The protocols for endometrial preparation were ultra‐long GnRH agonist, with decapeptide treatment for several months; however, no pregnancy was achieved after two ET cycles. Hysteroscopy had been performed before the last ET, but no specific problems had been reported.

## 3 INVESTIGATIONS

The couple was advised to refer to our infertility clinic by this time in order to try to conceive without gender selection. The infertility specialized team performed medical examinations on them. The standard sperm parameters of the male partner were normal (5th WHO criteria, 2010). FSH, LH, TSH, PRL, and AMH levels of the female partner were all normal. Demographic information is presented in Table 1. While menstrual cycles were regular, she complained of spotting in the middle of her menstrual cycle. She had no symptoms of galactorrhea and hirsutism. The uterine cavity and fallopian tubes were normal on the hysterosalpingography (HSG). The ultrasound measuring of the endometrium performed on the 5th day of menstruation showed the endometrial thickness of about 8 mm, along with the symptoms of uterine adenomyosis. In addition, a 20‐mm endometriotic lesion was observed in the left ovary.

**TABLE 1:** Clinical and demographic data of the couple

Female partner	
Age (year)	39
Secondary Infertility (year)	6
AMH (ng/µl)	2.5
FSH (IU/ml)	6.4
LH (IU/ml)	3.1
PRL (ng/dl)	15
TSH (µIU/ml)	1.5
Previous ICSI cycle	
Number of oocytes	15
Number of embryos	4
Current ICSI cycle	
Number of oocytes	12
MII oocytes	12
Number of frozen embryos	9

Male partner	
Age (year)	46
Sperm parameters	
Count (10^6^)	75
Morphology (%)	3
Motility (%)	
Progressive	51
Non‐progressive	22
Immotile	27

With a diagnosis of stage III/IV endometriosis, secondary infertility, and endometrial thickening, the patient was referred for laparoscopic surgery. Endometrial curettage and left ovarian cystectomy were performed. Ovarian endometrial cysts, proliferative endometrium, and functional uterine polyps were reported in pathology, but there was no endometritis.

Following laparoscopic surgery, the patient was given three to four months to conceive naturally, which she failed to achieve. The board of physicians decided to initiate ART cycle.

**FIGURE 1:**
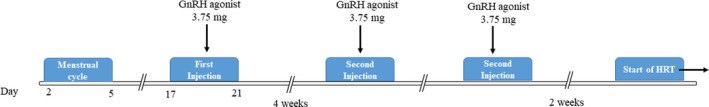
Schematic representation of long agonist protocol

Due to pelvic endometriosis and uterine adenomyosis, the ultra‐long downregulation protocol for a period of three months followed by a long agonist protocol was administrated prior to ART cycles. The patient received three doses of 3.75 mg Diphereline (every 28 days). Two weeks after the last dose, ovarian stimulation was started using 225 IU/day of Merional^®^ (hMG) and was continued until the day of hCG administration (11 days), and final oocyte maturation was triggered by Ovitrelle administration (250 micrograms equivalent to ~6500 IU hCG according to manufacturer data) (Figure 1).

During ovarian stimulation, endometrium was echogenic and contained secretions. For OHSS prevention, all embryos were frozen after ovarian pickup.

The ultra‐long downregulation protocol (Zoladex) was administrated prior to FET cycle for a period of two months. At the beginning of the FET cycle, the endometrium was 7 mm and irregular. Two weeks after the third dose of Zoladex, the thickness of the endometrium was 8 mm; therefore, the FET cycle was canceled. One month later, the patient received a dose of 3.75 mg of deferlin and then underwent another embryo transfer cycle, so HRT was started using estradiol valerate 6 mg. After the endometrial thickness reached 10.5 mm, a 50 mg progesterone was injected, and two good‐quality embryos (blastocyst stage) were transferred. The result of β‐hCG was negative after 16 days.

**FIGURE 2:**
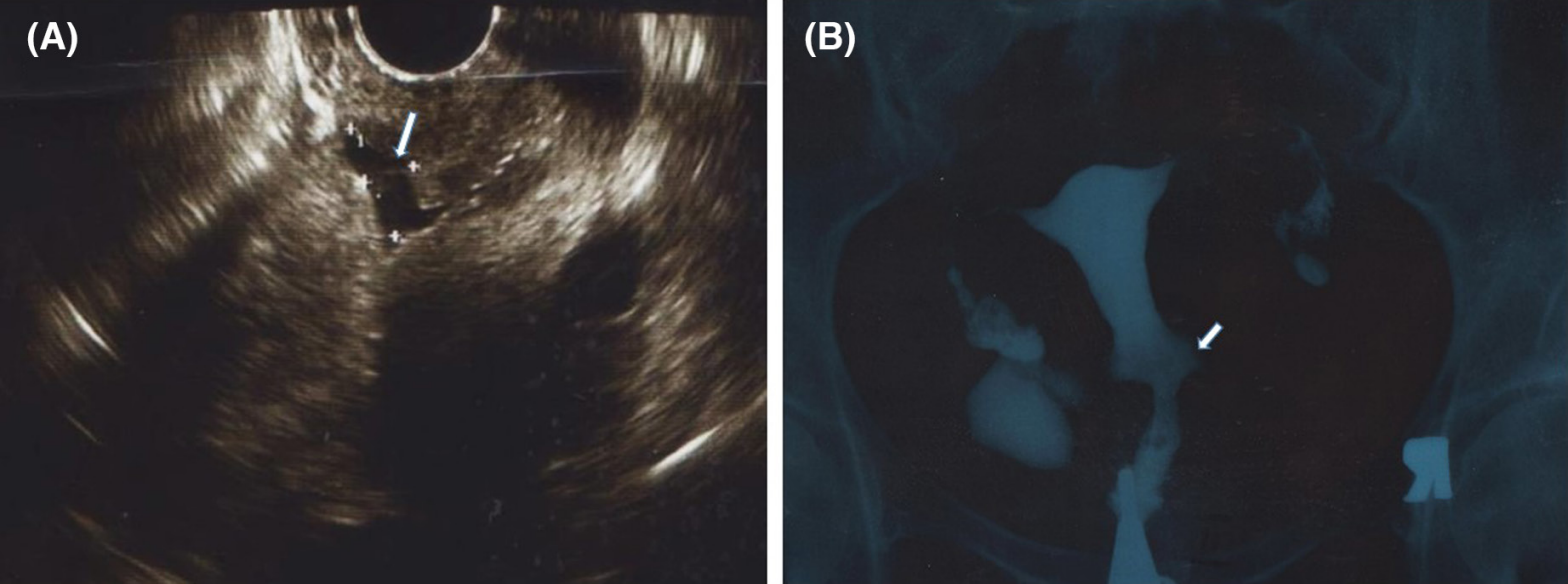
Isthmocele in the patient's uterine (A) The white arrow shows isthmocele on hysterosonography (B) The white arrow points to isthmocele's pouch on HSG

In order to ascertain the reason behind so many failed attempts, the female partner's uterine cavity was reassessed using 2D ultrasound of uterine, hysterosonography, and HSG. The 2D ultrasound revealed a 16 × 8 mm cesarean defect located in the lower segment of the anterior uterine wall. Hyperechogenic regions were seen in the cesarean defect areas, favoring the accumulation of secretions and bleeding. Hysterosonography of the uterus revealed adenomyosis, a 29 mm sub‐serosal fibroid in the posterior segment of uterus, and a 9 × 12 mm defect located in the lower segment of the uterus near the site of the cesarean scar. Thickness of residual myometrium over the defect was 4 mm and thickness of myometrium adjacent to the defect was 19 mm. In addition, isthmocele's pouch was revealed on HSG which further confirmed the diagnosis of isthmocele (Figure 2).

## 4 TREATMENT

According to the patient's previous history, which included an inappropriate endometrium pattern during the previous three embryo transfer cycles but a suitable thickness of the endometrium during ovarian stimulation or HRT protocol with ultra‐long GnRH protocol, and recurrent implantation failure in spite of good‐quality embryo transfer, as well as based on the recent hysterosonographic examination showing the signs of an isthmocele and discharge, a laparoscopic and hysteroscopic approach was selected to isthmocele resection and isthmoplasty before transferring the last patient's embryos.

Therefore, a skilled laparoscopic surgeon carefully trimmed the surrounding fibrotic tissue, removed it from the edges of the defect to access the healthy myometrium, and corrected the isthmocele. Later hysterosonography revealed that the area of previous isthmocele defect was reduced to 4 × 6 mm with no discharge. In addition, patient's blood spotting was reportedly gone following 2 months after surgery.

## 5 OUTCOME AND FOLLOW‐UP

At this time, FET by HRT was recommended to the patient. Fourteen days after 3.75 mg Diphereline injection, endometrial thickness was reported 8 mm. In the meantime, menstrual cycle had not yet begun; as a result, the β‐hCG level was checked, to test for spontaneous pregnancy, which was reported positive (20 mIU/ml). Therefore, the patient's FET cycle was canceled. However, lack of sufficient increase in β‐hCG resulted in abortion and failure to further proceed to clinical pregnancy. Because of this spontaneous pregnancy, the patient was given the chance to conceive naturally without any intervention for 6 months. Interestingly, only after three months, the patient became pregnant spontaneously, giving birth to a healthy baby boy.

## 6 DISCUSSION

Isthmocele is an underdiagnosed cause of secondary infertility. Increased knowledge and awareness of this common CS consequence prevents delays in diagnosis and reduces the burden on patients by avoiding other treatment approaches. Other more prevalent pathologies delayed isthmocele diagnosis in the initial evaluation of the presented case. Physicians should have more attention to the impact of isthmocele and have a closer look at identifying the mechanisms of isthmocele linking secondary infertility, implantation failure, and associated attributes.

Isthmocele discharge impedes or inhibits sperm motility, and the accumulation of secretions itself has a negative effect on embryo implantation. As a result, additional molecular research is needed to determine which grade of isthmocele causes these harmful effects and infertility. Several risk factors are suggested for incomplete healing of the uterine incision, including maternal body mass index, gestational diabetes, stage of labor, patient's age at CS, the extent of cervical dilatation at CS, and previous cesarean delivery.^7^, ^8^, ^9^ In light of how closely reproductive function, general health, and fertility are intertwined with isthmocele, we believe that now is an ideal time to resume research into identifying molecular mechanisms of isthmocele linking fertility and associated attributes. It is possible that certain patient‐related factors increase adhesion formation or impair wound healing. Furthermore, as isthmocele is a fluid‐filled pouch‐like defect, which resembles the hydrosalpinx condition,^10^ the immunological aspect of uterine endometrium is also an interesting research area as the accumulation of blood and fluid may be associated with infection and chronic endometritis^11^ which may lead to recurrent implantation failure. As a result, it would be advisable to assess the causes of chronic endometritis including isthmocele formation in cases of repeated implantation failure.

## AUTHOR CONTRIBUTIONS

Author 1: Elham Hosseini is the embryologist involved in drafting the initial manuscript, revising the manuscript, and approving the final submission. Author 2: Samaneh Aghajanpour involved in gathering patients' medical file data, revised the manuscript, and approved the final submission. Author 3: Nadia Zameni involved in editing the manuscript and approved the final submission. Author 4: Maryam Hafezi was the physician (gynecologist, fellowship in infertility) involved in patient care, revised the manuscript, and approved the final submission.

## CONFLICT OF INTEREST

All authors contributed substantially to the interpretation of the procedures in this case. Conflict of Interest. The authors declare no conflict of interest.

### CONSENT

Written informed consent was obtained from the patient to publish this report in accordance with the journal's patient consent policy.

## Data Availability

All data were mentioned in the manuscript.
